# Enterovirus D68 Infection in Children with Acute Flaccid Myelitis, Colorado, USA, 2014

**DOI:** 10.3201/eid2208.151949

**Published:** 2016-08

**Authors:** Negar Aliabadi, Kevin Messacar, Daniel M. Pastula, Christine C. Robinson, Eyal Leshem, James J. Sejvar, W. Allan Nix, M. Steven Oberste, Daniel R. Feikin, Samuel R. Dominguez

**Affiliations:** Centers for Disease Control and Prevention, Atlanta, Georgia, USA (N. Aliabadi, E. Leshem, J.J. Sejvar, W.A. Nix, M.S. Oberste, D.R. Feikin);; Children’s Hospital Colorado, Aurora, Colorado, USA (K. Messacar, C.C. Robinson, S.R. Dominguez);; Centers for Disease Control and Prevention, Fort Collins, Colorado, USA (D.M. Pastula);; University of Colorado Denver, Aurora (D.M. Pastula)

**Keywords:** myelitis, enterovirus infections, enterovirus-D68, acute flaccid paralysis, acute flaccid myelitis, viruses, children, Colorado, United States

## Abstract

During August 8, 2014–October 14, 2014, a total of 11 children with acute flaccid myelitis and distinctive neuroimaging changes were identified near Denver, Colorado, USA. A respiratory prodrome was experienced by 10, and nasopharyngeal specimens were positive for enterovirus D68 (EV-D68) for 4. To determine whether an association exists between EV-D68 infection and acute flaccid myelitis, we conducted a retrospective case–control study comparing these patients with 2 groups of outpatient control children (1 group tested for acute respiratory illness and 1 for *Bordetella pertussis *infection)*.* Adjusted analyses indicated that, for children with acute flaccid myelitis, the odds of having EV-D68 infection were 10.3 times greater than for those tested for acute respiratory infection and 4.5 times greater than for those tested for *B. pertussis* infection. No statistical association was seen between acute flaccid myelitis and non–EV-D68 enterovirus or rhinovirus infection. These findings support an association between EV-D68 infection and acute flaccid myelitis.

## Abstract 2

Odds of this viral infection in the nasopharynx were 10 times greater for children with this condition than for controls.

Enterovirus D68 (EV-D68) shares features with rhinoviruses (*1*) and primarily causes respiratory disease. Clusters of respiratory disease caused by EV-D68 have been reported in Asia, Europe, and the United States (*2*,*3*). Although EV-D68 has been identified in the central nervous system of 2 patients with limb weakness (*4*,*5*), its role in causing neuroinvasive disease has not been clearly defined.

From August 8, 2014, through October 14, 2014, a cluster of cases of acute limb weakness, cranial nerve dysfunction, or both, in children with characteristic radiologic findings of myelitis were identified at Children’s Hospital Colorado (CHCO), in Aurora, Colorado, USA. These cases represented a substantial increase over the number of children admitted with this same constellation of signs and symptoms in the previous 4 years at CHCO (*6*–*8*). This cluster prompted the Centers for Disease Control and Prevention (CDC) to create a case definition for acute flaccid myelitis (AFM; a subset of acute flaccid paralysis, characterized by appearance of myelitis on radiologic scans) and publish a national call for cases through a health alert announcement (*9*), which led to identification of cases nationally (*10*) and an additional case in Colorado.

The Colorado AFM cluster occurred during an outbreak of EV-D68 respiratory disease (*3*). During this period, CHCO emergency department visits and admissions for respiratory complaints to the hospital increased over prior years (*11*); EV-D68 detection among hospitalized patients subsequently increased (*7*). Although no etiology for the neurologic disease was identified among the Colorado cluster of patients (despite extensive testing, including metagenomic sequencing of cerebrospinal fluid), EV-D68 was found in the nasopharynx of 45% of these patients (*7*,*12*). We further investigated a possible epidemiologic association between EV-D68 and AFM by conducting a case–control study comparing the presence of EV-D68 in upper respiratory specimens of case-patients and 2 groups of control children. This analysis was determined by human subjects review at CDC and CHCO to be nonresearch and was conducted as a public health investigation.

## Methods

### Study Design and Setting

We conducted a retrospective case–control study of children who had received medical care for any illness necessitating collection of nasopharyngeal specimens for respiratory pathogen testing in Colorado during August 3, 2014–October 18, 2014 (the epidemiologic weeks when confirmed AFM cases were identified). AFM case-patients were defined as children <21 years of age who had acute neurologic illness characterized by focal weakness of >1 limbs, magnetic resonance imaging (MRI) findings of spinal cord lesions largely restricted to gray matter, and no identified etiology, per CDC case definition (*6*,*7*,*9*,*10*). 

We also identified 2 control groups of children for whom nasopharyngeal specimens had been obtained while they were CHCO outpatients during the study period. We selected outpatients as controls because most AFM case-patients had respiratory signs and symptoms and were evaluated as outpatients before neurologic symptoms developed. The first control group (respiratory pathogen panel [RPP]–tested controls) included children who were evaluated as outpatients and for whom nasopharyngeal specimens had been tested by multiplex RPP PCR (FilmArray; BioFire Diagnostics LLC, Salt Lake City, UT, USA), which detects adenovirus; coronaviruses HKU1, NL63, 229E, OC43; influenza viruses A(H1N1)pdm09, A(H3), and B; metapneumovirus, parainfluenza viruses 1–4; respiratory syncytial viruses A and B; enterovirus/rhinovirus; *Bordetella pertussis*;* Chlamydophila pneumoniae; *and *Mycoplasma pneumoniae*. The second control group (*B. pertussis *[BP]–tested controls) included children who were evaluated as outpatients and who had nasopharyngeal specimens obtained for PCR testing for *B. pertussis*. We excluded from the study infants <12 months of age and children >18 years of age because AFM patients in these age groups had not been identified in Colorado during this period. If multiple specimens from the same child were submitted for testing from different times during the study, only the first was included. Specimens submitted for RPP and BP testing from the same child and on the same date were considered for the RPP analysis only.

We also analyzed results of FilmArray testing for all patients admitted to the pediatric intensive care unit (PICU) at CHCO during July–November 2014. We chose this population because all patients admitted with respiratory symptoms routinely undergo FilmArray testing and results would provide a representative view of pathogens circulating in the community and resulting in severe respiratory illness at the time of the outbreak.

### Laboratory Testing

Nasopharyngeal specimens from AFM patients were initially tested by using the FilmArray panel, which has a sensitivity of 83.7% and a specificity of 100% for detecting enteroviruses and rhinoviruses but is unable to distinguish between them (*13*). Specimens positive for enterovirus/rhinovirus were sent to CDC for enterovirus viral protein (VP) 1 seminested reverse transcription PCR (RT-PCR) (*14*), followed by molecular sequencing of the VP1 amplicons. In October 2014, a new, highly sensitive, EV-D68 real-time RT-PCR (rRT-PCR) assay was developed at CDC (*15*), and samples from all AFM patients were also tested by using this assay. FilmArray testing was first conducted for the RPP-tested controls; specimens that were positive for enterovirus/rhinovirus were subsequently sent to CDC, along with all BP-tested specimens. At CDC, the RPP- and BP-tested control specimens were tested by using an rRT-PCR assay for pan-enteroviruses, which performs similarly to the VP1 seminested PCR assay (*16*). Specimens were also tested by rRT-PCR for EV-D68. All specimens positive by pan-enterovirus RT-rPCR that were not EV-D68 were also molecularly sequenced for virus identification. Samples from PICU patients were tested first with the FilmArray panel at CHCO; positive samples were sent to CDC, where they underwent the same series of testing as controls.

### Main Exposure and Covariates

The main exposure of interest was EV-D68 infection, defined as positive EV-D68 results obtained by rRT-PCR of a nasopharyngeal specimen. The second exposure of interest was infection with another enterovirus/rhinovirus, defined as a positive result by pan-enterovirus rRT-PCR but a negative result by EV-D68 rRT-PCR; that is, infection with any enteroviruses/rhinoviruses other than EV-D68. This group, referred to as enteroviruses/rhinoviruses excluding EV-D68, was chosen to provide a comparison with the EV-D68–positive group. Covariates included continuous variables (age, sex, days between symptom onset and collection of nasopharyngeal specimen, epidemiologic week of nasopharyngeal specimen collection) and categorical variables (fever [yes/no]; upper and/or lower respiratory symptoms including nasal congestion, rhinorrhea, sore throat, cough, wheeze, or respiratory distress [yes/no]; hospitalization for respiratory symptoms [yes/no]; and type of nasopharyngeal specimen obtained [swab/aspirate]). Although enterovirus detection in nasopharyngeal aspirates and nasopharyngeal swab samples is similar, more pathogens might be identified in nasopharyngeal swab samples (*17*). To ensure that no differences existed between groups with regard to this variable, we included it in our models.

### Statistical Analyses

Descriptive analyses reported characteristics of the AFM case-patients and control children. Medians and interquartile ranges were provided for age and days between symptom onset and collection of nasopharyngeal specimen because these variables were not normally distributed. Proportions were reported for categorical variables. The nonparametric Wilcoxon-Mann-Whitney 2-sample test was used to detect significant differences between continuous variables and between proportions for categorical variables. Those variables that differed significantly between AFM case-patients and controls (p<0.1) were included in the adjusted multivariable models, with the exception of epidemiologic week of sample collection, which was included in the adjusted models regardless of statistical significance. Models assessing AFM case-patients versus RPP-tested controls were adjusted for age, time between respiratory symptom onset and specimen collection, and epidemiologic week of specimen collection. Models assessing AFM case-patients versus BP-tested controls were adjusted for type of nasopharyngeal specimen, presence of fever, and epidemiologic week of sample collection. We report odds ratios, 95% CIs, and p values from logistic regression analyses in our models performed by using exact conditional analysis for small sample sizes. In the multivariable models, p<0.05 was considered statistically significant.

We tested 2 models for comparisons between AFM and each control group. Model 1 tested the association between EV-D68 and AFM; model 2 tested the association between enterovirus/rhinovirus excluding EV-D68 and AFM. To determine the sensitivity of our models among cases of neurologic weakness, which included acute cranial nerve deficits with or without limb weakness, we also conducted a sensitivity analysis among all patients in Colorado with acute neurologic weakness for whom nasopharyngeal specimens were available. Models and covariates used for this analysis were the same as those used in the main analysis. All analyses were performed with SAS statistical software package version 9.3 (SAS Institute, Inc., Cary, NC, USA).

## Results

**Figure 1:**
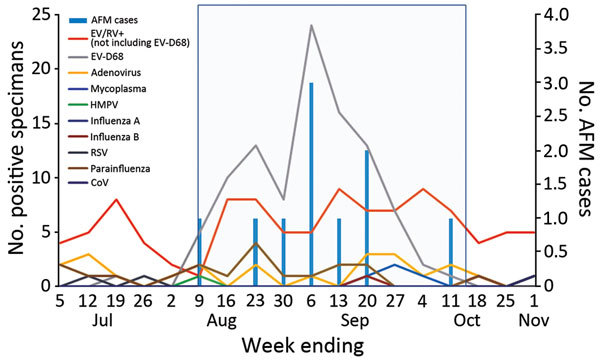
Pathogens isolated from patients with acute flaccid myelitis and from patients in a pediatric intensive care unit, Colorado, USA, July–November, 2014. Box indicates study period. AFM, acute flaccid myelitis; CoV, coronavirus; EV, enterovirus; HMPV, human metapneumovirus; RPP, respiratory pathogen panel; RSV, respiratory syncytial virus; RV, rhinovirus.

**Figure 2:**
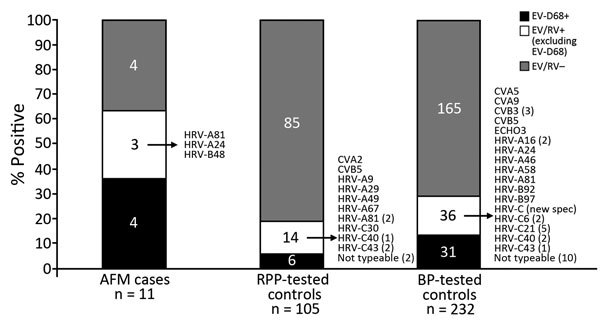
Results of enterovirus testing among case-patients and controls in study of acute flaccid myelitis, Colorado, USA, July–November, 2014. Arrows indicate specific strains identified in those specimens; numbers in parentheses indicate number of that type of strain. AFM, acute flaccid myelitis; BP, *Bordetella pertussis*; CV, coxsackievirus; echo, echovirus; EV, enterovirus; HRV, human rhinovirus; RPP, respiratory pathogen panel; RV, rhinovirus; RPP, respiratory pathogen panel.

The peak of AFM diagnoses coincided with the peak of EV-D68 respiratory infection detections at CHCO (*6*,*7*). Viral analyses of the specimens from PICU patients indicated that the predominant virus causing severe respiratory illness during the outbreak was EV-D68, followed by enterovirus/rhinovirus species excluding EV-D68 (Figure 1). Of the 203 specimens from PICU patients that were positive by RPP and sent to CDC for further testing, 100 (49%) were positive for EV-D68 (Figure 2). Among the other enterovirus/rhinovirus species, no predominant virus was in circulation. These species included human rhinoviruses, echoviruses, and coxsackieviruses A and B; some specimens could not be typed.

Overall, during the outbreak period in Colorado, we identified 13 patients who had acute neurologic disease with limb weakness, cranial nerve dysfunction, or both. Use of the CDC AFM case definition resulted in exclusion of 2 patients with acute neurologic disease who did not have limb weakness but who had cranial nerve deficits only. Of the remaining 11 patients, 10 were reported from CHCO and 1 was reported from another Denver metropolitan area hospital. From CHCO, we identified 105 RPP-tested controls and 232 BP-tested controls. All AFM patients had an antecedent acute illness; most (91%) reported respiratory illness (Table 1). 

**Table 1:** Characteristics of patients with acute flaccid myelitis and control patients, Colorado, August 3–October 18, 2014*

Characteristic	Case-patients, n = 11	Control patients			
		RPP-tested†	p value	BP-tested‡	p value
Sex, no. (%)					
M	8 (73)	62 (59)	0.52	124 (53)	0.24
F	3 (27)	43 (41)	NA	108 (47)	NA
Age, y, median (IQR, range)	8 (9, 1–18)	5 (8, 1–18)	0.05	7 (8, 1–18)	0.14
Respiratory symptoms, no. (%)	10 (91)	82 (79)§	0.69	221 (97)¶	0.25
Fever, no. (%)	10 (91)	79 (76)§	0.45	73 (32)#	<0.001
Hospitalized for respiratory symptoms, no. (%)	0	11 (11)**	0.60	12 (5)¶	1.00
Enterovirus testing, no. (%)					
EV/RV negative	4 (36)	85 (81)	NA	165 (71)	NA
EV/RV positive, excluding EV-D68	3 (27)	14 (13)	0.08††	36 (16)	0.12††
EV-D68 positive	4 (36)	6 (6)	0.02‡‡	31 (13)	0.03‡‡
Type of specimen, no. (%)					
Nasopharyngeal swab	6 (55)	57 (54)	1.00	192 (83)	0.04
Nasopharyngeal aspirate/wash	5 (45)	48 (46)	NA	40 (17)	NA
Time to specimen collection, d, median (IQR, range)§§	10 (7, 7–36)	5 (5, 0–31)¶¶	<0.001	14 (14, 1–120)##	0.91
Epidemiologic wk of specimen collection, median (range)	37 (33–42)	38 (32–42)	0.58	38 (32–42)	0.89

*BP, *Bordetella pertussis*; EV, enterovirus; IQR, interquartile range; NA, not applicable; RPP, respiratory pathogen panel; RV, rhinovirus. †Unless otherwise stated, denominator is 105 for RPP-tested controls.  ‡Unless otherwise stated, denominator is 232 for BP-tested controls. Denominators vary slightly because some observations were missing for covariates.  §n = 104.  ¶n = 227.  #n = 226. **n = 103.  ††p value comparing EV/RV-positives excluding EV-D68 with EV/RV negatives. ‡‡p value comparing EV-D68 with EV/RV-negatives. §§No. days between symptom onset and collection of nasopharyngeal specimen. ¶¶n = 102.  ##n = 222.

Comparing AFM case-patients with RPP-tested controls, we found that AFM case-patients were older (median age 8 years vs. 5 years, respectively; p = 0.05) and that specimens from AFM case-patients were collected later than specimens from RPP-tested controls (median 10 vs. 5 days after respiratory symptom onset, respectively; p<0.001). We found no statistically significant differences between these 2 groups with regard to sex, presence of upper or lower respiratory symptoms, presence of fever, hospitalizations for respiratory symptoms, type of specimen obtained, or epidemiologic week of specimen collection. 

Comparing AFM case-patients with BP-tested controls, we found that AFM case-patients were more often febrile (91% vs. 32%, respectively; p<0.001) and had fewer nasopharyngeal specimens collected by swab than by aspiration (55% vs. 83%, respectively; p = 0.04). We found no statistically significant differences between these 2 groups with regard to sex, age, presence of respiratory symptoms, hospitalizations for respiratory symptoms, timing of specimen collection, and epidemiologic week of specimen collection. Furthermore, epidemiologic week of specimen collection was not found to be a confounder (data not shown).

**Figure 3:**
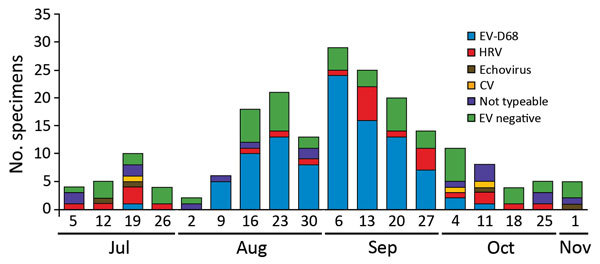
Sequencing results for 203 specimens from patients in a pediatric intensive care unit, Colorado, USA, 2014. All respiratory pathogen panel–positive samples were sent to the Centers for Disease Control and Prevention for further testing. Of these, 148 were positive by EV RT-PCR and 55 were negative by pan-EV RT-PCR. The 148 specimens positive by pan-EV RT-PCR were tested by EV-D68 real-time RT-PCR, and of these, 100 were positive (EV-D68). The remaining non–EV-D68 specimens were sent for molecular sequencing and were identified as 26 various HRVs, 4 echoviruses, and 3 CVs; 16 specimens were not typeable. One specimen was co-infected with HRV and CV. CV, coxsackievirus; EV, enterovirus: HRV, human rhinoviruses; RT-PCR, reverse transcription PCR.

Of the 11 AFM case-patients, 4 were infected with EV-D68, 4 were negative for enterovirus/rhinovirus according to pan-enteroviral RT-PCR, and 3 were positive according to pan-enteroviral RT-PCR; further typing of specimens from these 3 patients indicated a variety of rhinoviruses (Figure 3). One case-patient who was initially negative according to VP1 testing had a positive result on EV-D68 rRT-PCR, which was confirmed on repeat analysis. This discordance resulted from low EV-D68 RNA copy numbers in the specimen, at the limit of detection for both assays. The EV-D68 rRT-PCR cycle threshold for this specimen was 43.9 with a clear sigmoid curve. Given that we used the EV-D68 rRT-PCR for case-patients and controls, this patient was classified as EV-D68 positive. 

Among the AFM case-patients, characteristics did not differ among those positive and those negative for EV-D68 (data not shown). EV-D68 was detected more frequently in the nasopharyngeal specimens of AFM case-patients than in those of RPP-tested controls (36% vs. 6%, respectively; p = 0.02) and BP-tested controls (36% vs. 13%, respectively; p = 0.03) (Table 1). The non–EV-D68 enterovirus/rhinoviruses among the control groups included a mixture of coxsackieviruses, echoviruses, and rhinoviruses; some samples could not be typed. The odds of EV-D68 infection for AFM case-patients compared with RPP-tested controls were 10.3 (95% CI 1.8–64.8) when adjusted for age, days between respiratory symptoms and nasopharyngeal specimen collection, and the epidemiologic week of specimen collection (Table 2). The odds of EV-D68 infection for AFM case-patients compared with BP*-*tested controls were 4.5 (95% CI 1.0–21.2) when adjusted for the presence of fever, type of specimen collected, and epidemiologic week of specimen collection (Table 3). In this latter model, the odds of fever for AFM case-patients were nearly 19 times that for BP-tested controls. We found no association between enterovirus/rhinovirus excluding EV-D68 and AFM in comparison with either control group. Although epidemiologic week of specimen collection did not differ between groups (Table 1), this variable was further tested as a possible confounder in all models and did not meet criteria (data not shown).

**Table 2:** EV-D68 and EV/RV association with acute flaccid myelitis compared with RPP-tested controls, Colorado, USA, 2014*

Model	RPP-tested controls	
	Unadjusted models, OR (95% CI)	Adjusted models, OR (95% CI)
EV-D68	9.1 (1.9–42.0)†	10.3 (1.8–64.8)‡
Patient age	NA	1.0 (0.9–1.2)
Time to specimen collection	NA	1.1 (1.0–1.2)
Epidemiologic wk of specimen collection	NA	0.9 (0.7–1.3)
EV/RV excluding EV-D68	4.5 (0.8–23.9)§	6.9 (0.8–66.0)¶
Patient age	NA	1.1 (0.9–1.3)
Time to specimen collection	NA	1.1 (1.0–1.2)
Epidemiologic wk of specimen collection	NA	0.9 (0.6–1.2)

*Denominators vary slightly within models because some observations were missing for covariates. EV, enterovirus; NA, not applicable; OR, odds ratio; RPP, respiratory pathogen panel; RV, rhinovirus. †n = 105.  ‡n = 102. §n = 99.  ¶n = 96.

**Table 3:** EV-D68 association with acute flaccid myelitis compared with BP-tested controls, Colorado, USA, 2014*

Model	BP-tested controls	
	Unadjusted models, OR (95% CI)	Adjusted models, OR (95% CI)
EV-D68	3.7 (0.9–13.5)†	4.5 (1.0–21.2)‡
Fever	NA	18.9 (3.0–424.1)
Type of specimen	NA	0.2 (0.05–0.9)
Epidemiologic wk of specimen collection	NA	1.0 (0.8–1.3)
EV/RV excluding EV-D68	3.4 (0.6–17.2)§	2.5 (0.4–13.9)¶
Fever	NA	11.6 (1.6–276.5)
Type of specimen	NA	0.2 (0.04–1.2)
Epidemiologic wk of specimen collection	NA	1.1 (0.8–1.4)

*Denominators vary slightly within models because some observations were missing for covariates. BP, *Bordetella pertussis*; EV, enterovirus; NA, not applicable; OR, odds ratio; RV, rhinovirus. †n = 232.  ‡n = 226. §n = 201.  ¶n = 195.

### Sensitivity Analysis

We conducted a sensitivity analysis and included children who met the original Colorado case definition. This definition included 2 patients with cranial nerve dysfunction who did not meet the CDC AFM case definition criteria because they lacked acute limb weakness, compatible spinal MRI findings, or both (*7*). A nasopharyngeal specimen was collected from only 1 of these 2 patients and was positive for EV-D68, yielding 12 cases in this subanalysis. These results did not differ appreciably from those of the main analysis (Table 4).

**Table 4:** EV-D68 association with acute neurologic disease compared with that of RPP-tested and BP–tested controls, adjusted models*

Exposure	RPP-tested controls†			BP*-*tested controls‡	
OR (95% CI)	p value	OR (95% CI)	p value
EV-D68	12.9 (2.5–76.4)†	0.002		5.7 (1.3–25.5‡	0.02
EV/RV excluding EV-D68	6.9 (0.8–66.0)§	0.08		2.5 (0.4–13.9)¶	0.30

*Of 13 cases of neurologic disease identified in Colorado during the outbreak period, only 12 provided nasopharyngeal specimens for analysis. RPP models were adjusted for age, time to specimen collection, epidemiologic week of sample collection. *B. pertussis* models were adjusted for presence of fever, type of specimen collected, and epidemiologic week of specimen collection. BP, *Bordetella pertussis*; EV, enterovirus; OR, odds ratio; RPP, respiratory pathogen panel; RV, rhinovirus. †n = 102.  ‡n = 226. §n = 96. ¶n = 195.

## Discussion

Our study demonstrated an epidemiologic association between EV-D68 infection and AFM among children during the 2014 Colorado outbreak. The odds of EV-D68 infection were 10 times higher for children with AFM than for RPP-tested controls and 4.5 times higher than for BP-tested controls. The odds of fever were also higher for AFM case-patients than for BP-tested controls; this finding was not surprising, given the clinical picture associated with pertussis. The elevated odds of EV-D68 infection for AFM case-patients compared with RPP-tested controls suggest that the prevalence of EV-D68 infection among these AFM case-patients was not likely to reflect background circulation of the virus during the outbreak. Moreover, during the outbreak, this association seems to be unique to EV-D68 because infection with other enteroviruses, rhinoviruses, or other common respiratory pathogens identified through FilmArray was not significantly associated with AFM.

Age was a confounder in this analysis. RPP-tested controls were younger, reflecting the median 5 years of age reported during the 2014 EV-D68 respiratory outbreak (*18*). The older age of the AFM case-patients was similar to the median 7.6 years reported in the US description of AFM cases (*10*). However, data from Europe and Wales describe similar disease in younger children. In a report of 3 cases of neurologic dysfunction and laboratory evidence of EV-D68 infection in Europe, these patients were 4, 5, and 6 years of age (*19*,*20*). In addition, in Wales, the ages of a cluster of 4 children with acute flaccid paralysis (3 who had respiratory symptoms preceding the acute flaccid paralysis and 2 who were positive for EV-D68) was predominantly <2 years (*21*). These slightly discrepant data may be indicative of the small sample size of AFM cases and highlight the need for continued surveillance to better define the epidemiology of AFM cases.

The prevalence of EV-D68 in the nasopharyngeal specimens of the controls in our study (6%–13%) was lower than expected, given the common presence of EV-D68 in PICU patients. Although the rate of EV-D68 in communities during the 2014 US outbreak is unknown, data from other EV-D68 respiratory illness outbreaks in Asia and Europe suggest a similar low prevalence rate of EV-D68 positivity in nasopharyngeal specimens: 2.3%–10.9% among hospitalized children with respiratory symptoms (*2*,*22*–*25*) and 2.0% among outpatients with respiratory symptoms (*25*). The prevalence of EV-D68 among the controls was much lower than that seen among PICU patients, suggesting either a disproportionately high acuity of EV-D68 respiratory disease testing or selective testing by clinicians in the outpatient setting compared with the intensive care setting. As the outbreak was progressing, the official CHCO respiratory illness algorithm discouraged clinicians from testing all children with respiratory symptoms seen in emergency or outpatient settings for EV-D68 because the clinical management would not change for those who were treated as outpatients. As such, children with routine respiratory symptoms seen in the emergency room, urgent care, or other outpatient clinics were not being sampled for EV-D68, and children who were infected would have been missed. We tried to account for the potential decline in outpatient testing in 2 ways. First, although we did not find week of specimen collection to be a statistical confounder, we nonetheless included it in the multivariable model. Second, we chose an additional control group of children tested for *B. pertussis*. The clinical syndrome of pertussis in these children probably differed from the respiratory symptoms among children with acute respiratory illness, and the BP-tested children were probably sampled more systematically to rule out *B. pertussis *infection. These specimens were thus less likely to have a testing bias than were those from the RPP-tested control group. In the BP-tested controls, we saw a positive association between AFM case-patients and the presence of EV-D68.

A similar clinical presentation of some other picornaviruses lends biological plausibility to the association of EV-D68 and AFM. Enteroviruses such as enterovirus A71 (EV-A71) and poliovirus cause neurologic syndromes including acute flaccid paralysis, aseptic meningitis, and rhomboencephalitis. The MRI findings for the cluster of children in our study are similar to those induced by EV-A71 and poliovirus, both of which show tropism for the anterior horn cells of the spinal cord, although they infrequently infect the central nervous system (*26*,*27*). Similar to EV-D68, EV-A71 was initially linked to nonneurologic syndromes, specifically herpangina and hand, foot, and mouth disease, before outbreak data conclusively revealed an association between EV-A71 and neurologic syndromes. Other studies of the 2014 cluster of AFM cases have detected EV-D68 in the upper respiratory tract and, in 1 patient, in blood (*10*,*12*). However, EV-D68 is expected to be found at these sites in persons with EV-D68 respiratory illness, and detection of EV-D68 in these specimens does not prove causation of AFM. Identification of EV-D68 in cerebrospinal fluid, which provides the most definitive evidence of causation, was not reported from the 2014 cluster. Nonetheless, our study compares EV-D68 detection in AFM case-patients with detection in contemporaneous control patients with mild respiratory illness, lending additional epidemiologic support to the ecologic association between EV-D68 and AFM.

Our analysis was subject to several limitations. First, although an ideal control group would have included population-based sampling of all age-appropriate children in the Denver metropolitan area who did not have AFM during the outbreak, such a group was logistically not possible. Therefore, we used retrospective outpatient controls for whom RT-PCR diagnostic testing was performed at the discretion of providers at CHCO and specimens were retained and available for further testing. As such, our controls probably do not reflect all children in the community, and the sample might have been biased, representative only of children with mild respiratory symptoms not requiring hospitalization. The higher prevalence of EV-D68 among PICU patients suggests that prevalence in the community is higher than in the sample of children with mild respiratory symptoms for whom upper respiratory specimens were collected. However, given that most of the AFM case-patients were children with a mild respiratory prodrome, our control groups were more representative of the degree of respiratory illness seen in the case-patients than in PICU patients. Second, respiratory specimens were obtained after a much shorter interval from patients in the RPP-control group than from patients in the AFM case-patient and BP-control groups. This delay might have led to a lower prevalence of EV-D68 (or other pathogens) in these latter 2 groups than would have been found if testing had been performed sooner (*28*). Third, nasopharyngeal specimens are not sterile; presence of viruses in these samples might be coincidental and not causative of AFM. The association of the presence of EV-D68 in the nasopharynx and AFM might also have been biased by an unmeasured or unrecognized confounder. Fourth, RPP-negative specimens at CHCO were not sent to CDC for enterovirus/rhinovirus and EV-D68 rRT-PCR testing. Although sensitivity of the FilmArray assay is 83.7%, this test may have missed EV-D68–positive cases. Fifth, we also noted positive measures of association between non–EV-D68 enterovirus/rhinovirus exposure and AFM among both control groups, although neither association was statistically significant. However, this analysis did not have an adequate sample size to enable further exploration of this association. Last, although our models included a variable for the timing of specimen collection, because of the limited sample size of AFM case-patients we were unable to completely control for this variable through analyses that more closely matched with time of specimen collection (not shown).

In conclusion, we found an epidemiologic association between AFM and EV-D68 infection among children with respiratory illness during 2014 in Colorado. This finding goes beyond previously reported temporal associations between AFM clusters with increases in hospital admissions for respiratory symptoms and detection of EV-D68 in AFM case-patients. These epidemiologic data, combined with the biological plausibility of this association, suggest a possible causal link; however, a gap remains between the epidemiologic data and the data from extensive testing of laboratory specimens. CDC recommends continued surveillance, and a revised case definition without age restrictions has been implemented (*29*). For further investigation of this association, improved surveillance for AFM with timely and comprehensive specimen collection and testing for EV-D68 are needed.
